# Probing Red Blood Cell Membrane Microviscosity Using Fluorescence Anisotropy Decay Curves of the Lipophilic Dye PKH26

**DOI:** 10.3390/ijms232415767

**Published:** 2022-12-12

**Authors:** Alexey N. Semenov, Daniil A. Gvozdev, Anastasia M. Moysenovich, Dmitry V. Zlenko, Evgenia Yu. Parshina, Adil A. Baizhumanov, Gleb S. Budylin, Eugene G. Maksimov

**Affiliations:** 1 Faculty of Biology, M.V. Lomonosov Moscow State University, 1-12 Leninskie Gory Str., 119991 Moscow, Russia; 2 Laboratory of Clinical Biophotonics, Biomedical Science and Technology Park, I.M. Sechenov First Moscow State Medical University, 8-2 Trubetskaya Str., 119991 Moscow, Russia; 3 Interdisciplinary Scientific and Educational School, Molecular Technologies of the Living Systems and Synthetic Biology, M.V. Lomonosov Moscow State University, 1 Leninskie Gory Str., 119991 Moscow, Russia

**Keywords:** red blood cells membrane microviscosity, PKH26 lipophilic probe, time-resolved fluorescence anisotropy

## Abstract

Red blood cell (RBC) aggregation and deformation are governed by the molecular processes occurring on the membrane. Since several social important diseases are accompanied by alterations in RBC aggregation and deformability, it is important to develop a diagnostic parameter of RBC membrane structural integrity and stability. In this work, we propose membrane microviscosity assessed by time-resolved fluorescence anisotropy of the lipophilic PKH26 fluorescent probe as a diagnostic parameter. We measured the fluorescence decay curves of the PKH26 probe in the RBC membrane to establish the optimal parameters of the developed fluorescence assay. We observed a complex biphasic profile of the fluorescence anisotropy decay characterized by two correlation times corresponding to the rotational diffusion of free PKH26, and membrane-bounded molecules of the probe. The developed assay allowed us to estimate membrane microviscosity $\eta_{m}$ in the range of 100–500 cP depending on the temperature, which paves the way for assessing RBC membrane properties in clinical applications as predictors of blood microrheological abnormalities.

## 1. Introduction

Red blood cells (RBCs, erythrocytes) possess unique biomechanical properties that determine the microrheological behavior of blood. Reversible aggregation of RBC determines blood viscosity at low shear stress, and RBC deformability allows changing the cellular shape and squeezing into capillaries [1]. These intrinsic features of RBC govern the blood microcirculation in organs and tissues and partly define the efficiency of the transport function performed by blood [2]. Several pathologies are accompanied by disturbances in RBC aggregation and deformability. It was reported that diabetes mellitus types I and II [3,4,5,6], cardiovascular diseases, which include arterial hypertension [7,8], coronary heart decease [9], and particular cases of acute coronary syndrome [10] are all accompanied by alterations in blood rheology caused by deviations in RBC aggregation and deformability. Hemorheological abnormalities observed during chronic kidney disease [11] and specific cases of nephropathy [12] are suggested as manifestations of pathological increases in RBC aggregation and decreases in RBC deformability. In recent papers, it was demonstrated [13,14] that, in COVID-19 patients, RBC aggregation increased at both stasis and low shear rates, indicating subsequent disturbances in blood microcirculation and tissue perfusion, which serve as primary complication factors during rehabilitation and post-COVID syndrome. In [15], the authors, using contrast-enhanced ultrasound sonography (CEUS), revealed that arterial and venous organ perfusion of the liver, kidneys, spleen, and mesentery is affected due to abnormalities in the vascularization processes. In another recent paper, it was demonstrated [16] that COVID-19 significantly reduces the axial blood velocity in the capillary of the microcirculation system of the eye. Authors connected such pathology with massive microthrombosis, which can be related to erythrocytes microrheology disruptions, or possibly due to some unknown coagulation factor.

Knowledge of the molecular mechanisms of RBC aggregation and deformability is required to implement a proper and effective correction of deviations in RBC microrheological parameters. At present, these mechanisms are not understood completely due to the complexity of RBC interactions with each other, other blood cells, and vascular endothelium. Nowadays, both aggregation and deformability of RBCs are considered to be regulated by the molecular processes taking place on the cellular membrane [17]. There are different models of RBC aggregation. One of them suggests that the clustering of RBCs into aggregates is driven by the aggregation forces originating from the intermolecular interaction between residues of pro-aggregate macromolecules adsorbed on the cells’ membrane surfaces. This “bridging model” is actively used to describe RBC aggregation in environments containing various macromolecules, such as fibrinogen, immunoglobulins, and high-molecular-weight dextrans [18,19]. Different macromolecules, including blood plasma protein fibrinogen and serum albumin, are capable of adsorption onto the RBC membrane [20]. Usage of fluorescence microscopy combined with optical tweezers and microfluidics allowed visualizing the membrane adsorption of dextran 70 kDa [21] and fibrinogen [22], pointing out that the adsorption is partially specific and is implemented by glycoprotein receptor complexes. This fact is of particular interest for clinical hemorheology as it suggests novel tools for the correction of increased fibrinogen-induced RBC aggregation by inhibiting the sites of macromolecule-specific recognition. However, such actions demand keeping the state of the membrane of the RBC intact as changes in membrane properties may significantly affect the erythrocytes’ transport function. Therefore, the parameter characterizing the membrane state is required to establish the proper treatment.

The striking flexibility of RBCs is realized by the unique structure of its cytoskeleton and the complexes of the membrane proteins responsible for the connection of the cytoskeleton to the inner surface of the membrane. The RBC cytoskeleton consists mostly of actin and spectrin microfilaments, which form a mash-alike net with its joints associated with the membrane via specific anchoring protein complexes, such as “band 3–p4.2–ankyrin” and “glycophorin C–p4.1–p55” [23]. Residues of the proteins comprising these complexes can be phosphorylated upon G-protein coupled receptors signaling, and the activation of ionic channels or membrane enzymes [24]. After phosphorylation, the protein molecules change the conformation, leading to the reversible disintegration of the whole complex, and the release of cytoskeleton joints from the membrane with subsequent support for deformability. The adenylyl cyclase signaling cascade utilizes protein kinase A phosphorylation upon the G-coupled receptor activation and serves as a potent target for a selective increase in the RBC deformability in the cases when it is impaired [25].

The interrelation between RBC aggregation and deformability has been studied for some time. In [26], the authors measured different parameters of both RBC deformation and aggregation in a variety of physiological and pathological conditions. They observed a weak yet clear correlation between aggregation and deformability in healthy donor erythrocytes. The analogous result was observed in the recent study [27] when the RBC deformability was impaired artificially using glutaraldehyde and/or osmolarity changes. Interestingly, the interrelation between aggregation and deformation of RBCs almost disappears in the case of membranopathies of erythrocytes occurring in some severe hereditary diseases. Yet, there is no complete understanding of the underlying mechanism. One mechanism supposes eruptions of membrane proteins associated with junctional cytoskeleton complexes with consequent influence on RBC membrane fluidity and viscoelasticity. For example, in [28], in the McLeod syndrome, the absence of the XK-protein, which is associated with the “spectrin–actin–band 4.1” complex, leads to the abnormal phosphorylation of the joint proteins with consequent destabilization of the cytoskeletal network resulting in the generation of acanthocytes. In [29], a similar result was supported by the experimental data on the observed eruptions in the phosphorylation of several proteins, including band 3, which takes place in neuroacanthocytosis. In [26], D. Lazari et al. suggested that described disturbances in the association between cytoskeleton and membrane affect deformation more than aggregation, thereby reducing the correlation between both processes. It is unknown whether the membrane phospholipids are affected directly during these complicated processes, and how the membrane fluidity and its viscoelastic properties are affected. Several computational studies of the RBC membrane, such as J. Gounley and Y. Peng, 2015 [30] and D.A. Fedosov et al. 2018 [31] propose that corresponding interrelation mechanisms are possible. From this viewpoint, experimental techniques for measuring membrane fluidity are required to study these processes in detail.

Many new methods, especially utilizing rapidly developing microfluidic technologies, are being presented nowadays to assess the parameters of RBC aggregation and deformation [32,33,34]. However, the problem of characterizing the RBC membrane during the measurements is out of scope in most modern techniques. Membrane microscopic viscosity (microviscosity) can be proposed as a diagnostic parameter of RBC membrane stability and integrity. Cellular membrane microviscosity was found to be sensitive to the early stages of several pathologies. Microviscosity was used as a parameter in modern oncology to predict possible malignization of the tumor cells [35] or to characterize the response and resistance of cancer cells to chemical therapy [36]. Changes in membrane microviscosity were used to track the effects of oxidative stress and assess neuroprotection activity in neuronal cells [37]. Several techniques allow measuring membrane microviscosity: Raman spectroscopy, which quantifies the gauche/trans conformational ratio of fatty acids [38]; electron spin resonance spectroscopy, which utilizes reporter molecules sensitive to the microenvironment (spin probes) [39]; fluorescence recovery after photobleaching [40]. The aim of the present study was to assess RBC membrane fluidity microviscosity by measuring the time-resolved fluorescence anisotropy of the non-specific lipophilic probe PKH26 bounded by the erythrocyte membrane. To verify the obtained results, we performed the measurements of temperature-dependence of microviscosity of model egg yolk lecithin liposomes, labeled with the same fluorescent probe. The obtained results allowed us to propose the developed fluorescence assay as a novel tool for measuring membrane physiological intactness, which can be used in clinical applications to study RBC membrane properties at hemorheological disorders.

## 2. Results

### 2.1. Laser Confocal Microscopy Measurements of RBC, Stained with PKH26 Fluorescent Probe

**Figure 1:**
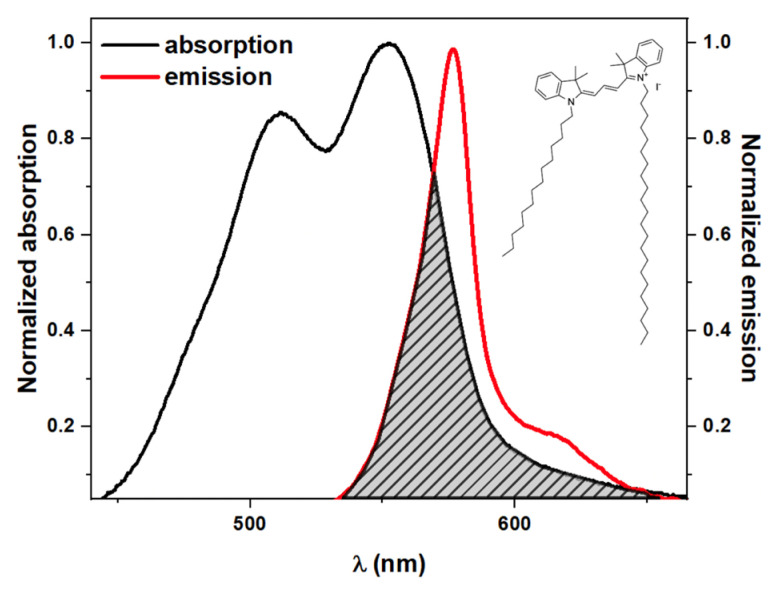
Chemical structure, absorption, and emission spectra of the PKH26 probe.

In the present work, we used PKH26, which is a highly fluorescent, non-specific lipophilic long-chain carbocyanine dye used to stain artificial and biological membranes [41]. Aliphatic tails of PKH26 molecules (the chemical structure is presented in Figure 1) allow to rapidly incorporate into the exposed lipid bilayer, establishing non-covalent interactions [42].

**Figure 2:**
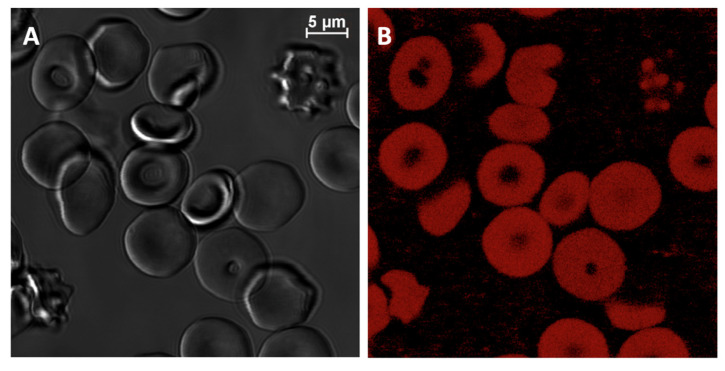
Transmission and confocal fluorescent microscopy imaging of RBCs stained by lipophilic fluorescence dye PKH26; (**A**) transmitted light image; (**B**) laser confocal image (λ_exc_ = 532 nm; λ_det_ = 567 nm).

First, we implemented conventional confocal microscopy of the RBCs, stained with PKH26, to prove the binding of PKH26 by the RBC membrane. Staining of RBCs for the confocal microscopy was performed using the protocol implemented in [43]. The total concentration of PKH26 during the staining was 2 μM. The representative transmitted light microscopic image and corresponding laser confocal microscopy image of the RBCs stained with PKH26 are presented in Figure 2.

### 2.2. Measurements of Time-Resolved Fluorescence of PKH26 Probe Bounded by RBC

PKH26 absorption and emission spectra are shown in Figure 1. One can observe the significant overlap between the absorption and emission spectra of PKH26. It means that in the case of the close proximity of probe molecules, which can be achieved at a high concentration of PKH26, numerous non-radiative transfers driven by the FRET mechanism would take place affecting anisotropy decay. Aggregation and oligomerization of PKH26 molecules, which is also possible at a high concentration of the probe, may result in static quenching of the detected fluorescence. In this case, it would require longer exposition or higher irradiation power to observe comparable levels of fluorescence intensity. Thus, the choice of PKH26 concentration should be thoroughly adjusted.

Time-resolved fluorescence measurements required for the fluorescence anisotropy assessment were performed using a custom-built set-up utilizing an experimental cuvette containing 1 mL of blood suspension in PBS at 0.1% hematocrit. In accordance with the time-resolved fluorescence anisotropy measuring technique (details are provided in Section 4.5), total fluorescence intensity decay curves $I\left(t\right)$ of PKH26-labeled RBC samples were measured at different PKH26 concentrations. The obtained fluorescence decay curves $I\left(t\right)$ were approximated with the double-exponential decay function. The mean amplitude-weighted lifetime (*τ_mean_*) was calculated as follows:

(1) $$\tau_{mean}=\frac{A_{1}\tau_{1}+A_{2}\tau_{2}}{A_{1}+A_{2}}.$$

$A_{1},\tau_{1},A_{2},\tau_{2}$ are the corresponding parameters of the double-exponential function approximation of the total fluorescence intensity.

**Figure 3:**
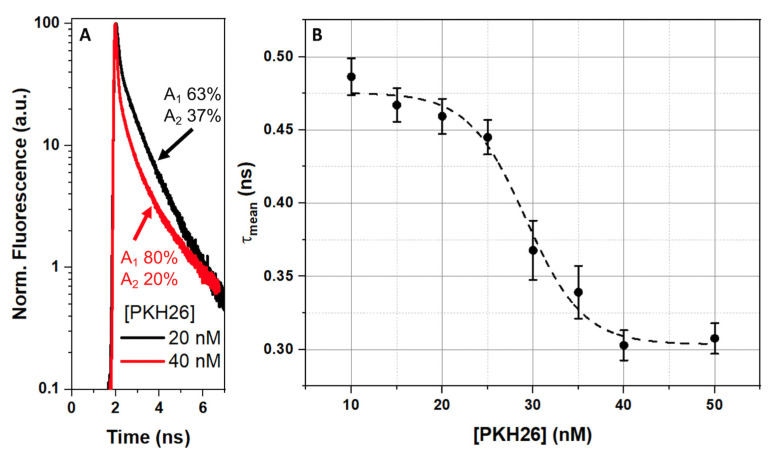
Time-resolved fluorescence measurements of RBC, labeled with PKH26 lipophilic dye at different concentrations: (**A**)—normalized on the maximum total fluorescence intensity decay curves; (**B**)—mean amplitude-weighted fluorescence lifetime (*τ_mean_*) values. Amplitudes A_1_ and A_2_ were obtained by approximation of the curves with a double-exponential decay function. Dashed line represents approximation with a sigmoid function.

The results of the measurements of the time-resolved total fluorescence intensity of the PKH26 concentration in the range of 10 to 50 nM are presented in Figure 3. The analysis of fluorescence decays at 20 nM and 40 nM PKH26 concentrations (Figure 3A) revealed a significant redistribution between the amplitudes of fast and slow decay components with the decay becoming faster at higher PKH26 concentrations. The role of the concentration-dependent effect on *τ_mean_* is demonstrated in Figure 3B: the PKH26 concentration in RBC suspension increased, *τ_mean_* monotonously decreased from 486 ± 12 ps (at [PKH26] = 10 nM) to 445 ± 11 ps (at [PKH26] = 25 nM) with a rapid reduction to 360 ± 20 ps at [PKH26] = 30 nM. Therefore, the usage of a high PKH26 concentration would lead to inevitable alterations in the corresponding anisotropy decay. Considering this, we proposed 20 nM as the optimum for the PKH26 concentration for fluorescence anisotropy measurements. Since concentration-driven effects influence fluorescence anisotropy as well, regardless of the membrane type, we suggest using the same PKH26 concentration for studying either RBC samples or liposomes.

Additionally, at a 20 nM concentration of PKH26, fluorescence in the sample of the stained RBCs significantly exceeds the signal of non-labeled intact RBCs, which are considered to originate from hemoglobin photoproducts [44]. Supplementary Figure S1 demonstrates the fluorescence decay curves of PKH26-stained RBCs (black curve) and non-labeled intact RBCs (green curve) showing the difference between intrinsic RBC fluorescence and PKH26 fluorescence bounded by the cellular membrane.

### 2.3. Anisotropy Decay of PKH26 Fluorescent Probe within the Membranes of RBCs and Egg Yolk Lecithin Liposomes at Different Temperatures

**Figure 4:**
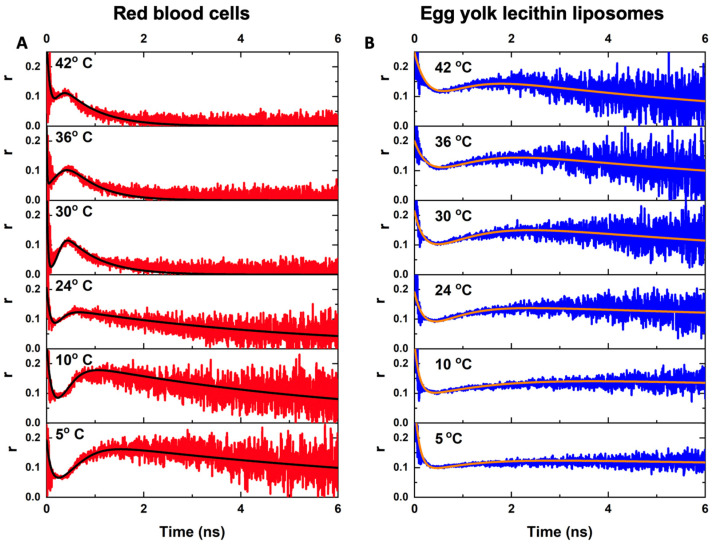
Anisotropy decay curves $r\left(t\right)$ of lipophilic fluorescence dye PKH26 in membranes of (**A**) red blood cells; (**B**) egg yolk lecithin liposomes, measured at different temperatures. [PKH26] = 20 nM. Black and orange lines represent the applications of associated decay approaches for anisotropy decay fitting..

Figure 4 demonstrates anisotropy decay curves $r\left(t\right)$ of PKH26 in membranes of RBCs (Figure 4A) and egg yolk lecithin liposomes (Figure 4B) at different temperatures measured at a PKH26 concentration of 20 nM. One can observe complex biphasic kinetics of anisotropy decay. A qualitative analysis of these curves allowed us to distinguish two regions on the time scale: the initial region (<1 ns) and the remaining one (>1 ns). The initial region was very fast and almost did not change with the temperature. In the case of PKH26-labeled RBCs, at high temperatures (42 °C), during the first 500 ps, anisotropy decreased to a lesser degree than at lower temperatures. Such behavior might be explained by the fact that the permeability of the RBC membranes with PKH26 molecules was more effective at higher temperatures. Whereas, anisotropy of the PKH26 probe embedded into egg yolk lecithin liposome membranes in this initial region was not temperature-dependent to the same extent.

The behavior of anisotropy decay in the remaining region (>1 ns) was temperature-dependent for both RBCs and egg yolk lecithin liposomes. It is clearly seen that as the temperature increased, the relaxation time of the anisotropy decay in this region decreased. It means that the rotational diffusions of the PKH26 molecules were significantly affected since the membrane microviscosity decreased. The changes in the >1 ns region were more pronounced in the RBCs than in the egg yolk lecithin liposomes.

The associated decay approach (Equation (5)) was implemented to fit anisotropy decay curves of PKH26 in the membranes of RBCs and egg yolk lecithin liposomes at different temperatures (Figure 4) to characterize the curves. As a result, we obtained values of multiple correlation times, $\theta_{1}$ and $\theta_{2}$ (Table 1), which correspond to the biphasic profile of measured anisotropy decay curves, and originated from the occurrence of two fractions of PKH26 molecules during the measurements, $\theta_{1}$ << $\theta_{2}$, and, thus, can be related to the rotational diffusion of the free unbounded PKH26 molecules fraction. The anisotropy decay in the initial region was fast because the rotational movements of free PKH26 molecules in the solution were fast enough even at low temperatures. That is the reason why the temperature dependence of $\theta_{1}$ was not observed. The correlation time $\theta_{2}$ corresponded to the fitting of anisotropy curves in the >1 ns region, and the temperature dependence was clearly pronounced (the corresponding graphs are presented in Supplementary Figure S2A). Based on these results, we can suggest that $\theta_{2}$ corresponds to the rotational diffusion of the membrane-bounded PKH26 molecule fraction and, thus, can be used to determine RBC membrane microviscosity. Assuming the PKH26 fluorescent probe rotation size of approximately 2–3 Å from the estimations of the distance between the heterocycles in carbocyanine dyes [45], and inserting the experimentally obtained values of $\theta_{2}$ considered as the membrane-bounded fraction correlation time into Equation (2), the membrane microviscosity ($\eta_{m}$) was estimated for membranes of RBCs and egg yolk lecithin liposomes at different temperatures. The values are presented in Table 1, and are also illustrated in Supplementary Figure S2B.

## 3. Discussion

The non-specific nature of PKH26 interaction with membranes governed the constitution of two populations of PKH26 probes with different rotational diffusion properties resulting in the complex biphasic profile of anisotropy decay curves (Figure 4). The occurrence of these two populations of the same single probe can be generally driven by several mechanisms. One possible mechanism suggests that different PKH26 molecules are bounded by different sites on the membrane. Another mechanism suggests that these fractions can be described as free-unbounded, and membrane-bounded PKH26 molecules. This case seems preferable since the initial region time $\theta_{1}$ is very short and, thus, reflects the fast rotation of the free unbound molecule in the solution.

The usage of liposomes in the present study confirmed this mechanism of multiple correlation times, $\theta_{1}$ and $\theta_{2}$. The membrane microviscosity of fresh egg yolk lecithin liposomes was higher than the microviscosity of RBC membranes and slightly higher (Table 1 and Supplementary Figure S2) than the membrane fluidity microviscosity of typical lecithin liposomes [46]. We previously obtained similar results of high membrane microviscosity of fresh egg yolk lecithin-made liposomes in comparison with liposomes prepared from different phospholipids [47]. Such a result can be explained by the abundance of additional components, which, along with phosphatidylcholine (80.5%) and phosphatidylethanolamine (11.7%) as main constituents, are presented in fresh egg yolk lecithin, and include cholesterol, lysophosphatidylcholine, sphingomyelin, and several minor neutral lipids [48]. Moreover, membrane microviscosity, which is measured by the rotational diffusion parameters of the probe, significantly depends on the depth of the localization of the probe within the bilipid layer, and generally needs to be determined [49]. It means that, in the case of a non-specific fluorescent probe, molecular dynamics simulations are required to characterize its localization within the membrane in order to increase the precision of the microviscosity assessment. The ratio between fractions of free (characterized with $\theta_{1}$) and membrane-bounded (characterized with $\theta_{2}$) PKH26 molecules is defined by the quality of the staining procedure. The staining should be more stable at high concentrations of PKH26 in the range of 2–4 μM, as postulated in the staining protocols, and is suitable for conventional fluorescence confocal microscopy. However, such large concentrations would lead to inevitable alterations in the fluorescence parameters due to possible self-quenching mechanisms, significantly complicating the correct interpretation of the experimental data on anisotropy decay. Overpassing this obstacle would demand either higher irradiation power or longer irradiation exposition during the measurements, which, in the cases of living cell studies, can be unacceptable due to possible phototoxic manifestations. Another limitation of the study is that it greatly depends on the optimal conditions for the fluorescent measurements of a particular fluorescent probe. PKH26, which was at our disposal, is a very common fluorescent probe that finds its application in many areas, including labeling RBCs for hematological studies. However, its application for measuring membrane fluidity by fluorescent anisotropy is complicated with the difficulties in the definition of its sizes and peculiarities of its interactions with the phospholipid membrane. More studies involving different probes, including molecular rotors, are needed to establish criteria for an optimal choice of the fluorescent probe.

The data presented in Table 1 correspond to the RBC membrane microviscosity related to the fluidity of membrane phospholipids. Considering the instrumental error, the obtained values of RBC membrane microviscosity were found to be of the same order of magnitude according to the data available in the literature. In [50], it was demonstrated that the enrichment of RBC membrane with cholesterol affects phospholipid fluidity, resulting in a significant increase in the RBC membrane microviscosity in the range from 100 to 600 cP, depending on the temperature. This result was achieved by assessing the rotational diffusion of the diphenylhexatriene (DPH) probe, and our experimental results, obtained by measuring time-resolved fluorescence anisotropy of the PKH26 probe, are in good agreement. RBC membrane fluidity viscosity was also measured using electron paramagnetic resonance (EPR) spectroscopy [51,52].

Membrane microviscosity may significantly vary for different cells depending on their physiology and functionality. For example, the microviscosity of the membranes of epithelium cells or human colorectal carcinoma cells was assessed by FLIM using the molecular BODIPY-based rotor and was estimated in the range of 400–500 cP, depending on the type of the implemented chemotherapy [53]. The fluidity of phospholipids of RBC membranes was found to be higher, which can be related to the necessity of erythrocytes in supporting the optimal rheological conditions for the blood flow.

## 4. Materials and Methods

### 4.1. Blood Sampling and RBC Extraction

A volume of 10 μL of blood from a single healthy male donor was sampled by the finger-prick method using a sterile lancet and placed into 1 mL of phosphate-buffered saline (PBS, pH 7.4 1×, Gibco). The obtained suspension was centrifuged (Eppendorf MiniSpin, Hamburg, Germany) at 3000 rpm for 1.5 min to sediment RBC. After the sedimentation, RBCs were extracted and washed three times in PBS (3000 rpm, 3 min). All manipulations with blood were performed in accordance with the general guidelines for hemorheological laboratory techniques [54].

### 4.2. Liposomes Synthesis

Liposomes were synthesized from fresh egg yolk lecithin (20 mg of lecithin was dissolved in 1 mL of chloroform) using the protocol described in [55] with minor modifications using the freeze–thaw extrusion method. The obtained suspension contained liposomes with a mean diameter of 100 ± 10 nm (as assessed by dynamic light scattering technique using Zetasizer Nano ZS (Malvern Instruments, Ltd., Malvern, Great Britain)).

Egg yolk lecithin liposomes were used as the model membrane object to establish the mechanisms of the complex shape of anisotropic decay $r\left(t\right)$ curves (Figure 4). Liposomes were studied under exactly the same conditions as RBCs, except that their concentrations were 10 times higher since the liposomal mean diameter was significantly less than that of RBCs.

### 4.3. Confocal Microscopy

The confocal microscopy imaging was performed using an Eclipse Ti-E microscope with confocal module A1 (Nikon Corporation, Tokyo, Japan) with a 532 nm excitation and detection at 567 nm.

### 4.4. Time-Resolved Fluorescence Anisotropy, Absorption, and Emission Spectroscopy Measurements

To assess fluorescence anisotropy, the corresponding fluorescence decay curves with picosecond time resolutions were collected by a time-correlated single photon counting (TCSPC) custom-built set-up comprised of a single photon counting module SPC-130EM paired with a hybrid detector HMP-100-40, with the power supply controlled by DCC-100 (all made by Becker&Hickl, Berlin, Germany). The sample excitation was performed with vertically polarized light at 510 nm using a picosecond laser (InTop, St.-Petersburg, Russia) with a 26 ps pulse duration driven at a repetition rate of up to 25 MHz. A long-pass filter with a 550 nm wavelength (FEL0550, Thorlabs, Newton, NJ, USA) was used to cut off the excitation signal. We thoroughly adjusted all optomechanical blocks in our experimental set-up to avoid the impact of light scattering on the detection. Detection was performed at 590 nm using monochromator ML-44 (Solar Laser Systems, Minks, Belarus). The polarization was adjusted by ultra-broadband wire-grid polarizers (WP25M-UB, Thorlabs, Newton, NJ, USA). The volume of the experimental sample in the cuvette was 1 mL. All measurements were performed in a temperature-control cuvette holder Qpod 2e with magnet-based stirring (Quantum Northwest, Liberty Lake, WA, USA).

Absorption and emission spectra measurements were performed using the Flame UV-VIS spectrometer (Ocean Insight, Orlando, FL, USA). Absorption spectra measurements involved illumination using a fiber-coupled white light source SLS201L/M (Thorlabs, Newton, NJ, USA).

### 4.5. Assessment of Microviscosity Using Time-Resolved Fluorescence Anisotropy

PKH26 (PKH26 Red Fluorescent Cell Linker Kit PKH26GL, Sigma Aldrich, Merck, Darmstadt, Germany) was used as a fluorescent probe to measure the membrane microviscosity of RBCs and egg yolk lecithin liposomes. The stock solution of the fluorescent probe PKH26 was diluted with PBS to achieve the desired concentrations and was put into the experimental cuvette in 1 mL volume. Prior to measurements, 1 μL of washed RBCs (to achieve 0.1% hematocrit) or 30 μL of egg yolk lecithin liposome solution (to achieve 0.6 mg/mL lipid-based concentration) were added directly to the experimental cuvette with PKH26-solution, incubated for 15 min at 20 °C and then measured.

Membrane microviscosity $\eta_{m}$ was measured by assessing time-resolved anisotropy of the fluorescence of PKH26, docked in membranes of RBC or liposomes, using equations [56,57,58]:

(2) $$\eta_{m}=\frac{\theta \times 3K_{B}T}{4\pi R^{3}},$$

where θ—rotational correlation time, *K_B_*—Boltzmann constant, *T*—temperature, and *R*—size of the fluorescent probe.

The rotational correlation time θ can be obtained from the analysis of the anisotropy decay curve $r\left(t\right)$. The physical principle describing the connections between θ and $\eta_{m}$ is illustrated in Supplementary Figure S3. When the sample in the cuvette is irradiated with polarized light (in our set-up vertical polarization was used), the occurring fluorescence includes light originating from photo-selected chromophores (which dipoles coincide (correlate) with the polarization plane of the incident light and, thus, the emission is also vertically polarized) and non-polarized emission, originating from chromophores, in which the dipole is not correlated with the polarization of the incident light due to rotational diffusion. Fluorescence originating from photo-selected oriented chromophores $I_{\left|\right|}\left(t\right)$ is detected by the vertically positioned polarizer located in front of the detector. The fluorescence originating from non-photo-selected chromophores $I_{⊥}\left(t\right)$ is detected by the horizontally positioned polarizer in front of the detector. Anisotropy decay $r\left(t\right)$ is calculated by confronting fluorescence decay curves $I_{\left|\right|}\left(t\right)$ and $I_{⊥}\left(t\right)$ is in accordance with the formula:

(3) $$r\left(t\right)=\frac{I_{\left|\right|}\left(t\right)-GI_{⊥}\left(t\right)}{I_{\left|\right|}\left(t\right)+2GI_{⊥}\left(t\right)},$$

and describes the process of the depolarization of the emitted fluorescence, i.e., the transition of photo-selected chromophores from the ordered to the randomized state due to rotational diffusion, G—the factor of the difference in the detection sensitivity to vertical and horizontal light polarization. Total fluorescence intensity is calculated as a sum of $I_{\left|\right|}\left(t\right)$ and $I_{⊥}\left(t\right)$:

(4) $$I\left(t\right)=I_{\left|\right|}\left(t\right)+2GI_{⊥}\left(t\right).$$

The characteristic time of anisotropy decay $r\left(t\right)$ is called the rotational correlation time θ. Since rotational diffusion depends on the microviscosity, the anisotropy decay strongly depends on the viscosity of the environment, in which the chromophores are located. It means that the characteristic time of anisotropy decay describes how fast the process of depolarization is, or, in other words, the velocity of the rotational movements of the molecule.

Estimation of the rotational correlation time is performed by fitting $r\left(t\right)$ with the multi-exponent decay function. In the case of a complex configuration of chromophores fractions (observed in our study (Figure 4) when a single chromophore was in a different environment and, thus, performed different rotational motions), multiple correlation times are possible. In this way, the anisotropy decay can be fitted using the associated decay approach [59,60,61]. For two distant fractions of chromophores, the associated decay formula will be:

(5) $$\tilde{r}\left(t\right)=\frac{{\tilde{A}}_{1}e^{-\frac{t}{{\tilde{\tau }}_{1}}}r_{01}e^{-\frac{t}{\theta_{1}}}+{\tilde{A}}_{2}e^{-\frac{t}{{\tilde{\tau }}_{2}}}r_{02}e^{-\frac{t}{\theta_{2}}}}{{\tilde{A}}_{1}e^{-\frac{t}{{\tilde{\tau }}_{1}}}+{\tilde{A}}_{2}e^{-\frac{t}{{\tilde{\tau }}_{2}}}},$$

where the fitting parameters ${\tilde{\tau }}_{1},{\tilde{\tau }}_{2},{\tilde{A}}_{1},{\tilde{A}}_{2}$ describe the fluorescent lifetime properties of each fraction, and $r_{01},r_{02}$ describe the steady-state anisotropy corresponding to each fraction. The parameters of the total fluorescence intensity decay $I\left(t\right)$ approximation, provided in Equation (1), can be used as rough estimations of the corresponding fitting parameters for the associated decay approach. All of these parameters are sorted to establish the best quality of the fitting of the experimentally measured anisotropy decay. The control of the quality of the approximation was based on the minimum of the reduced χ^2^ and tracking the coefficient of determination (COD), which was controlled by the proximity of R^2^ to 1 during the fitting. Thus, the obtained correlation times, $\theta_{1}$ and $\theta_{2}$, describe the rotational diffusion of a single type of chromophore in two different environments. In order to evaluate the microviscosity of each environment, one should use the corresponding correlation time with subsequent calculations using Equation (2).

**Figure 5:**
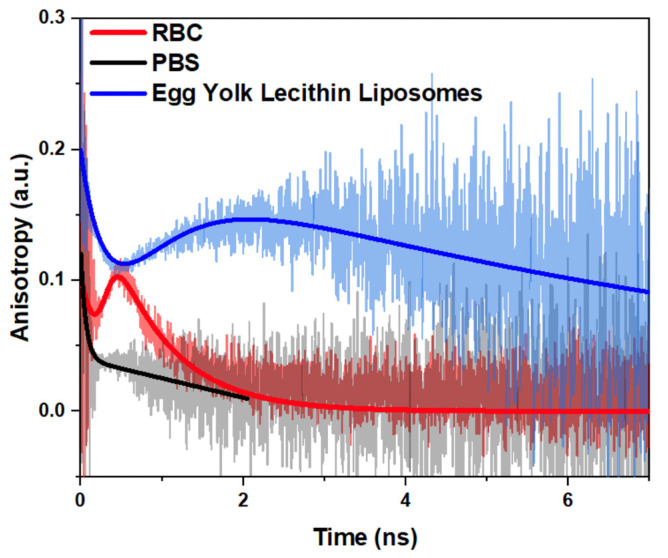
Analysis of anisotropy kinetics of lipophilic fluorescence dye PKH26 in the membranes of RBC, egg yolk lecithin liposomes, and phosphate-buffered saline (PBS) using the associated decay approach. Measurements were performed at 36 °C. Concentration of PKH26 = 20 nM; hematocrit 0.1%; liposomes sample represented 1%-suspension.

Figure 5 demonstrates, in detail, the implementation of the associated decay approach to analyze anisotropy decay of the fluorescence of the PKH26 probe in the RBC membrane (red curve) and egg yolk lecithin liposomes membrane (blue curve) at the same PKH26 concentration (20 nM), at 36 °C. The anisotropy decay curve of the fast freely-rotating PKH26 in PBS (black curve) was characterized by the double-exponential decay approximation. The initial (<1 ns on the time scale) region’s correlation time, $\theta_{1}$, was very short and did not change significantly between samples and, thus, corresponded to free rotations of unbound molecules of the probe. The remaining (>1 ns) region was characterized by a longer correlation time, $\theta_{2}$, which in RBC and the liposomes was in the nanoseconds range and, thus, was considered to correspond to the rotations of membrane-bounded PKH26 molecules. Comparing RBCs and egg yolk lecithin liposomes, $\theta_{2}$ was higher in liposomes than in RBC, demonstrating that egg yolk lecithin liposomal membranes were more viscous than RBC membranes.

## 5. Conclusions

Among other techniques to measure microviscosity, time-resolved fluorescence anisotropy has its own advantages, such as an opportunity to study samples dissolved in an aqueous environment. Since blood is a unique type of tissue, which is constantly flowing, RBCs are the most abundant fractions of blood cells, and their biomechanical and microrheological properties determine microcirculation, the task of assessing the microviscosity of erythrocyte membranes is of significant interest; as our study shows, blood cell suspension can be assessed by time-resolved anisotropy methods. Deformability and aggregation of RBCs are extremely important integral microrheological parameters since they have high impacts on the viscosity of the whole blood [13,16,62]. RBC deformability and aggregation depend on the properties of the cellular membrane. It was demonstrated [63] that the fixation of RBCs with glutaraldehyde led to a significant decrease in RBC deformability accompanied by an increase in the effective viscosity of the lipid membrane. The authors suggested it as a dominant factor dictating the dynamic responses of RBCs in pressure-driven flows. From this viewpoint, membrane microviscosity can be considered an indicative parameter of RBC membrane stability and integrity and, therefore, a novel microrheological parameter of the blood. Further studies of RBC membrane microviscosity of various diseases and under the effects of different agents are necessary to determine its contribution and sensitivity to the pathologies of the blood’s rheological behavior regulation.

The time-resolved fluorescence anisotropy technique allows for measuring the microviscosity of RBC membranes labeled with a fluorescent probe. However, to support the high quality of the obtained results, the probe should satisfy several criteria, namely, absorption and emission properties should support the minimal probability of the excited state annihilation and self-quenching mechanisms; and the lipophilic character of the interaction with membranes should ensure stable and sustainable labeling. Fulfilling these conditions would allow to significantly increase the accuracy of the time-resolved fluorescence technique, which might open up new potentialities of applications of such methods in clinical hematology to study blood cell membrane alterations that accompany various socially important diseases.

## Figures and Tables

**Table 1:** Correlation times $\theta_{1}$ and $\theta_{2}$, and membrane microviscosity ($\eta_{m}$) of RBCs and egg yolk lecithin liposomes assessed at different temperatures by studying the anisotropy of PKH26 fluorescence. Anisotropy curves were analyzed by the associated decay approach; $\theta_{1}$ corresponds to the rotational diffusion of free unbound PKH26, $\theta_{2}$ corresponds to the rotational diffusion of membrane-bounded PKH26 molecules.

Temperature ℃		5	10	24	30	36	42
RBC	$\theta_{1}$ (ns)	0.13 ± 0.03	0.14 ± 0.04	0.14 ± 0.03	0.11 ± 0.03	0.11 ± 0.04	0.16 ± 0.04
	$\theta_{2}$ (ns)	8.5 ± 1.3	6.0 ± 0.8	4.3 ± 0.5	1.7 ± 0.4	1.5 ± 0.4	1.7 ± 0.3
	$\eta_{m}$ (cP)	490 ± 140	350 ± 110	310 ± 90	110 ± 30	97 ± 29	94 ± 28
Egg lecithin liposome	$\theta_{1}$ (ns)	0.15 ± 0.02	0.16 ± 0.02	0.16 ± 0.03	0.17 ± 0.02	0.16 ± 0.02	0.17 ± 0.05
	$\theta_{2}$ (ns)	46 ± 7	35 ± 5	25 ± 3	11 ± 3	8 ± 2	6 ± 2
	$\eta_{m}$ (cP)	2740 ± 420	2110 ± 290	1610 ± 180	690 ± 190	560 ± 150	450 ± 150

## Data Availability

Not applicable.

## Supplementary Materials

The following supporting information can be downloaded at: https://www.mdpi.com/article/10.3390/ijms232415767/s1.
