# Supplementary material for: Probing Red Blood Cell Membrane Microviscosity Using Fluorescence Anisotropy Decay Curves of the Lipophilic Dye PKH26
Source: Int J Mol Sci. 2022 Dec 12;23(24):15767. doi: 10.3390/ijms232415767 (PMC9781149; doi:10.3390/ijms232415767)
Supplement: Supplementary file 1 [file ijms-23-15767-s001.zip › ijms-2049807-supplementary.pdf]

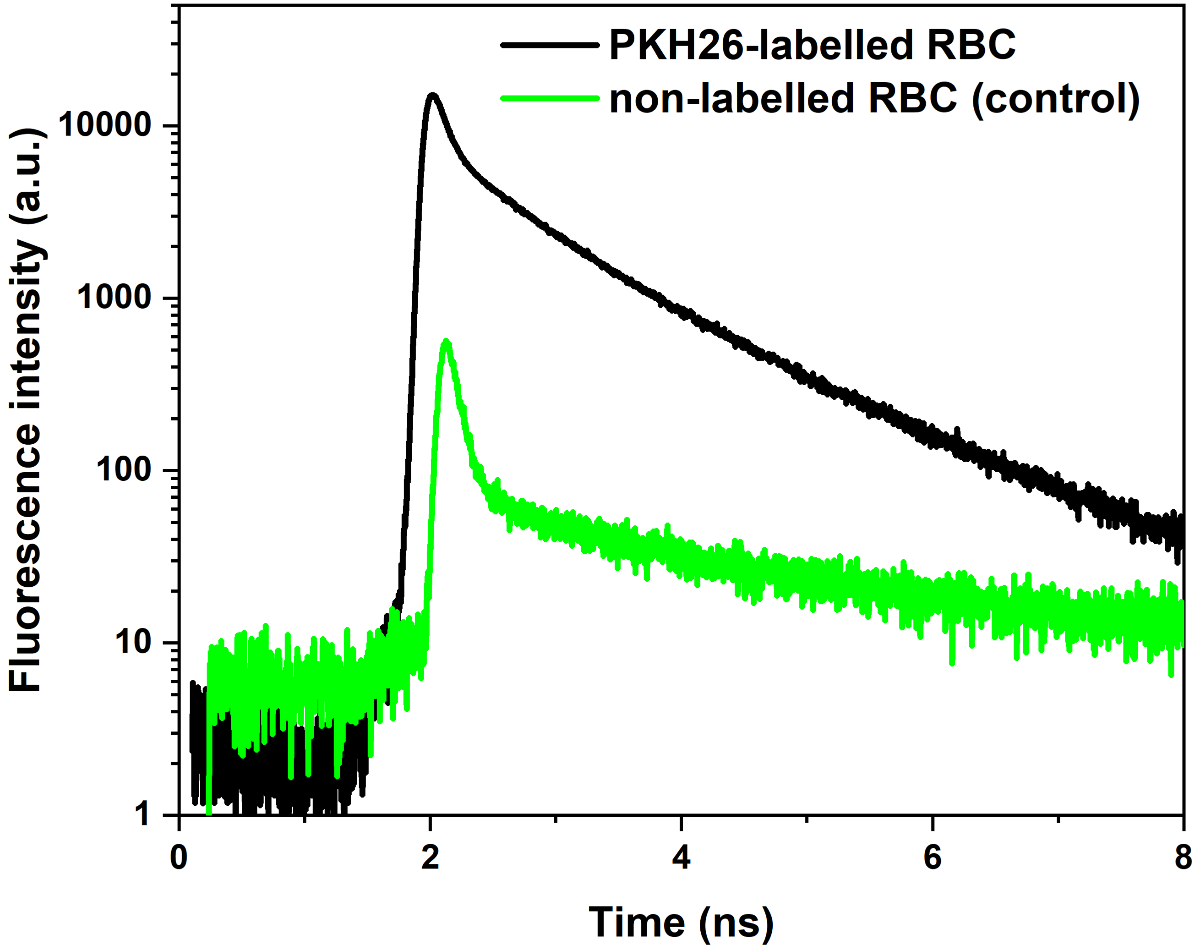

Supplementary Figure S1. Fluorescence decay curve of PKH26-labelled ([PKH26] = 20 nM) and non-labelled (control) RBC. λ_{exc} = 510 nm, λ_{det}=590 nm. One can see that at 20 nM concentration of PKH26 fluorescence in the sample of the stained RBC sample significantly exceeds the signal of non-labeled intact RBC. This signal originates from the formation of hemoglobin photoproducts which is responsible for two-photon and single photon-excited fluorescence of red blood cells (Shirshin E.A., Yakimov B.P. et al. doi: 10.1088/1612-202X/aac003).

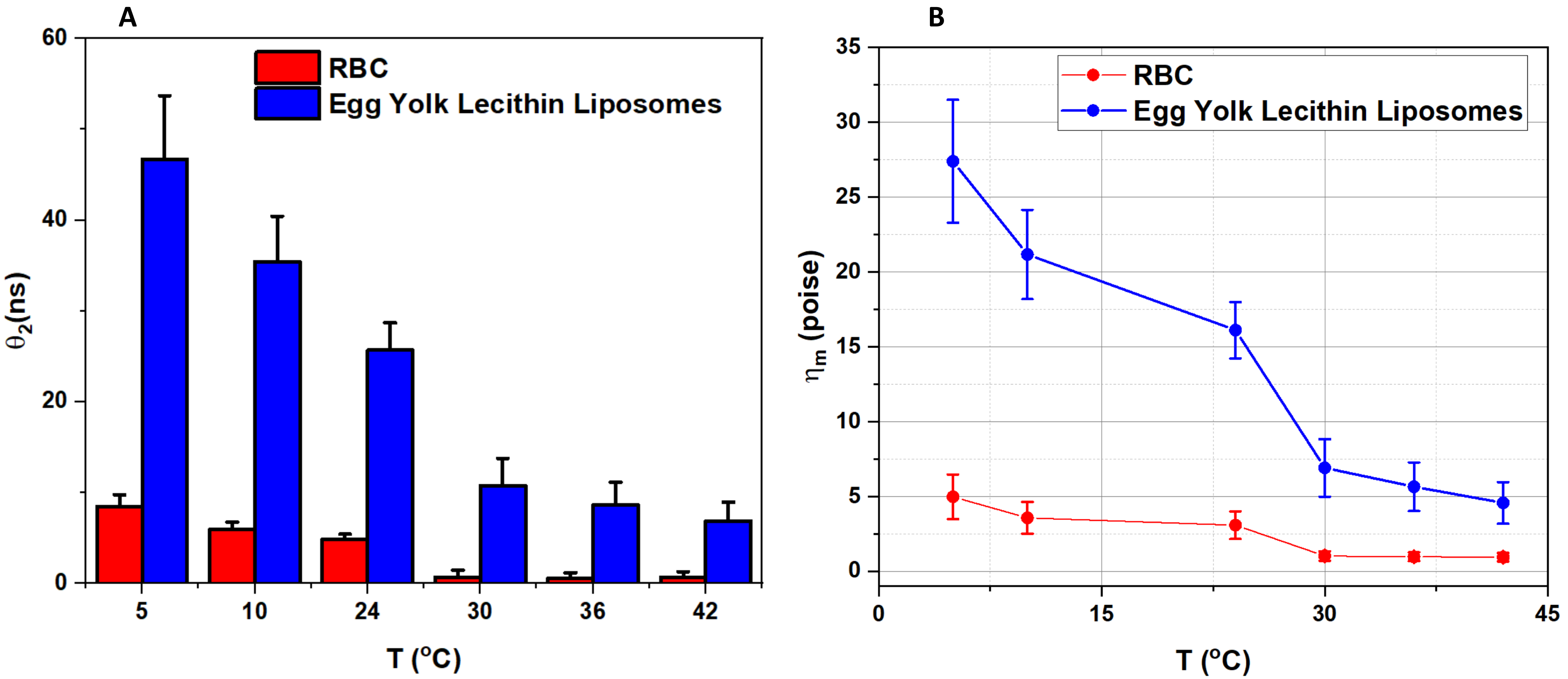

Supplementary Figure S2. The dependence of (A) - correlation time θ_2 of PKH26 fluorescence anisotropy and (B) - the microviscosity η_m of membranes of egg yolk lecithin liposomes and red blood cells obtained from the rotational diffusion of fluorescent probe PKH26, embedded into membrane, basing on its time-resolved fluorescence anisotropy. [PKH26] = 20 nM. Hematocrit 0.1%. Liposomes represent 1%-suspension.

Supplementary Figure S3. Physical basis of measuring microviscosity using time-resolved fluorescence anisotropy (step-wise description). (1) the solution is irradiated with polarized light; (2) the occurring fluorescence will include: signal originated from photo-selected chromophores (those, which dipole momentums coincide (correlate) with polarization plane) and signal originated from chromophores with another orientation of dipoles due to rotational diffusion in the solution; (3) Detection of polarized emission ($I_{||}$) and non-polarized emission ($I_{\perp}$).

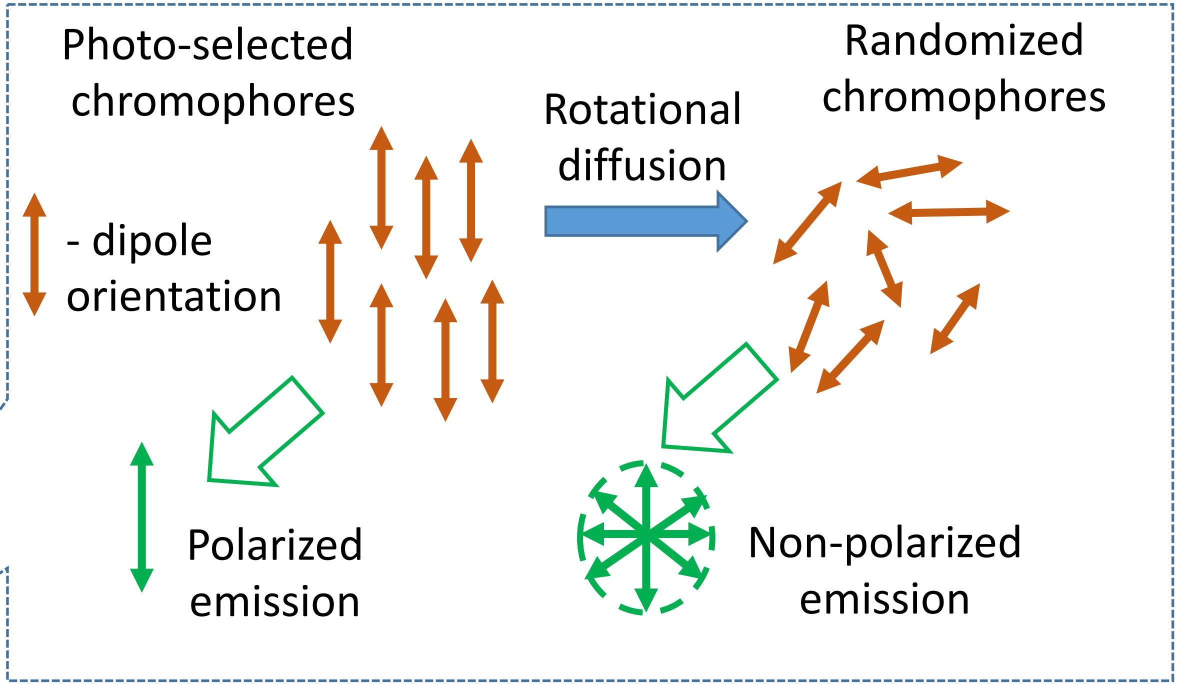

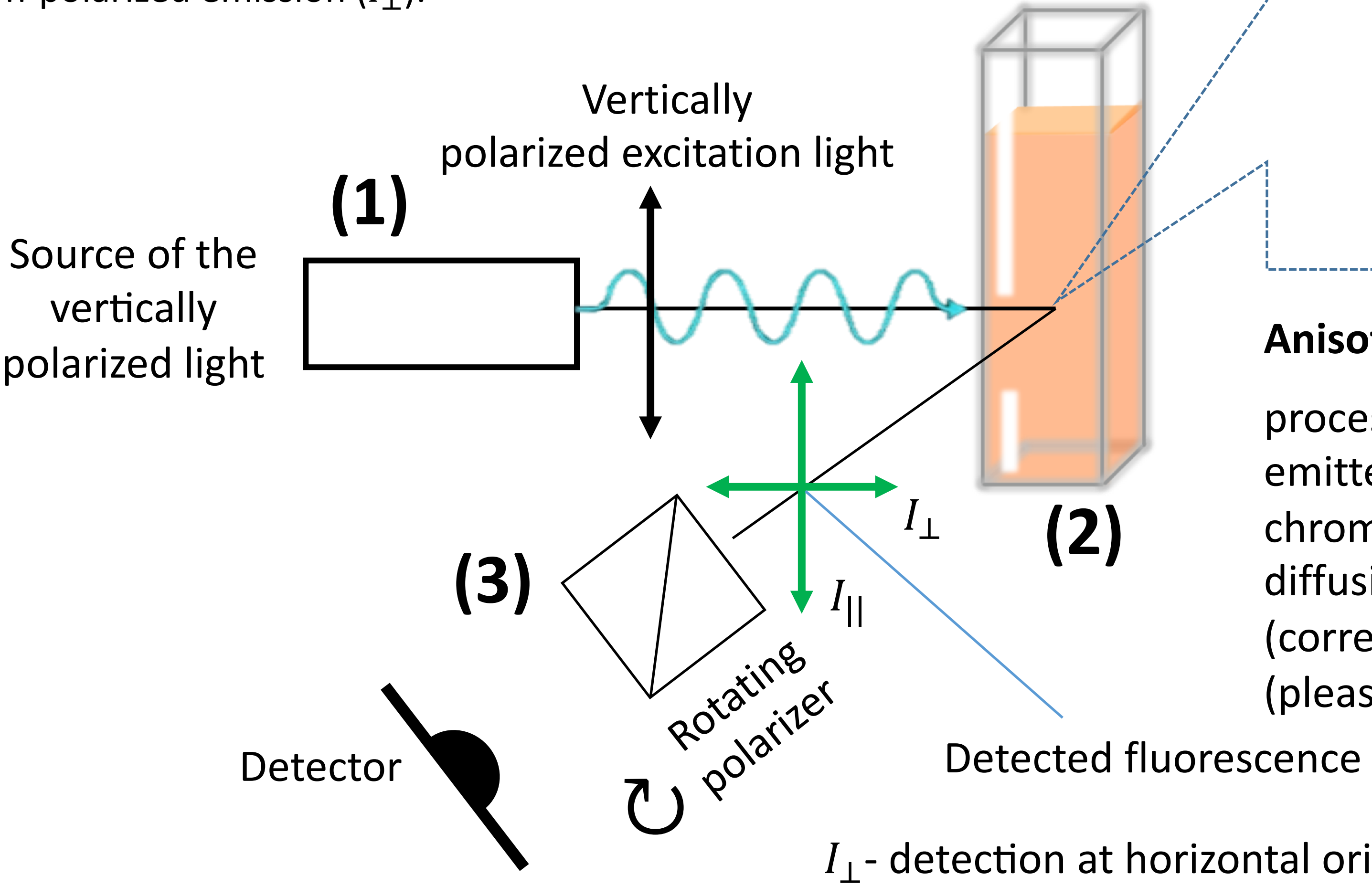

Anisotropy kinetics $r(t) = \frac{I_{||}(t) - GI_{\perp}(t)}{I_{||}(t) + 2GI_{\perp}(t)}$ describes the process of the depolarization (decorrelation) of the emitted fluorescence, i.e. transition of photo-selected chromophores to the randomized state due to rotational diffusion. The characteristic time of this transition (correlation time θ) depends on the microviscosity (please refer to Perrin equation for more details).

$I_{\perp}$ - detection at horizontal orientation of the polarizer
$I_{||}$ - detection at vertical orientation of the polarizer
